# Fibroblast transition to an endothelial “trans” state improves cell reprogramming efficiency

**DOI:** 10.1038/s41598-021-02056-x

**Published:** 2021-11-19

**Authors:** Megumi Mathison, Deepthi Sanagasetti, Vivek P. Singh, Aarthi Pugazenthi, Jaya Pratap Pinnamaneni, Christopher T. Ryan, Jianchang Yang, Todd K. Rosengart

**Affiliations:** Michael E. DeBakey Department of Surgery, Baylor College of Medicine, Houston, TX USA

**Keywords:** Cell biology, Cardiology

## Abstract

Fibroblast reprogramming offers the potential for myocardial regeneration via in situ cell transdifferentiation. We explored a novel strategy leveraging endothelial cell plasticity to enhance reprogramming efficiency. Rat cardiac endothelial cells and fibroblasts were treated with Gata4, Mef2c, and Tbx5 (GMT) to assess the cardio-differentiation potential of these cells. The endothelial cell transdifferentiation factor ETV2 was transiently over-expressed in fibroblasts followed by GMT treatment to assess “trans-endothelial” cardio-differentiation. Endothelial cells treated with GMT generated more cTnT^+^ cells than did cardiac fibroblasts (13% ± 2% vs 4% ± 0.5%, p < 0.01). Cardiac fibroblasts treated with ETV2 demonstrated increased endothelial cell markers, and when then treated with GMT yielded greater prevalence of cells expressing cardiomyocyte markers including cTnT than did fibroblasts treated with GMT or ETV2 (10.3% ± 0.2% vs 1.7% ± 0.06% and 0.6 ± 0.03, p < 0.01). Rat cardiac fibroblasts treated with GMT + ETV2 demonstrated calcium transients upon electrical stimulation and contractility synchronous with surrounding neonatal cardiomyocytes, whereas cells treated with GMT or ETV2 alone failed to contract in co-culture experiments. Human cardiac fibroblasts treated with ETV2 and then GMT likewise demonstrated greater prevalence of cTnT expression than did cells treated with GMT alone (2.8-fold increase, p < 0.05). Cardiac fibroblast transitioning through a trans-endothelial state appears to enhance cardio-differentiation by enhancing fibroblast plasticity.

Since the first demonstration a decade ago of the possibility of cardiac cellular reprogramming, a wide variety of transcription factors, microRNAs and chemicals have been shown to induce the transdifferentiation of cardiac fibroblasts into “induced cardiomyocytes” (iCMs)^1–9^. Despite encouraging observations that cardiac transdifferentiation improves post-infarct cardiac function in small animal models, limits on the efficiency of this process, especially in human cells, has catalyzed the search for more effective cardiac reprograming strategies^10–14^. Evidence that epigenetic repression of gene activation in higher order species may underlie cell resistance to reprogramming suggests that enhancing cell *plasticity*—the susceptibility of cells to transdifferentiation – may represent a promising strategy for enhancing cell reprogramming efficacy^6, 14, 15^.

Endothelial cells possess the capacity to undergo a native cell transdifferentiation process termed endothelial mesenchymal transition (EndMT), which is characterized by enhanced cell plasticity^16–19^. During development, endothelial cells likewise share a common mesodermal progenitor with cardiomyocytes. We therefore hypothesized that endothelial cells may represent a “plastic” cell target more conducive to cardio-differentiation than are fibroblasts. Given however the relative scarcity of endothelial cells compared to fibroblast in the infarct milieu, we theorized that it would be desirable to transdifferentiate fibroblasts into endothelial cells as our primary reprogramming target, and then use these cells as a substrate for the application of our cardio-differentiating factors. In this report, we describe the efficacy of this strategy using the vascular endothelial cell master regulator ETS variant 2 (ETV2) to induce an endothelial cell “trans-state,” which in turn enhanced our ability to generate iCMs from cardiac fibroblasts^20–23^.

## Methods

### Cell and vectors

All animal experiments were approved by Institutional Animal Care and Use Committee (IACUC) at Baylor College of Medicine and all methods were carried out in accordance with the NIH guidelines (Guide for the care and use of laboratory animals) and under protocol AN-6223, approved by the IACUC. These studies were conducted and are reported in compliance with relevant elements of ARRIVE guidelines.

Commercially procured rat cardiac microvascular endothelial cells (AS One International Inc., SantaClara, CA) were cultured on fibronectin-coated dishes in EGM-2 medium supplemented with 10 ng/ml VEGF and bFGF (Lonza, cc-3156, cc-3162). Cardiac fibroblasts were isolated from adult rat cardiac tissues harvested from 6- to 8-week-old Sprague–Dawley rats (Envigo International Holding Inc., Hackensack, NJ) using standard cell explant protocols, and cultured in DMEM, 10% fetal bovine serum (FBS), and 1% penicillin/streptomycin. Human cardiac fibroblasts (PromoCell GmbH, Heidelberg, Germany) were cultured in Medium 106 (Gibco, ThermoFisher) supplemented with low serum growth supplement kit (S003K, Gibco, ThermoFisher).

Lentivirus vectors encoding ETV2 tagged with yellow fluorescent protein (Venus), or encoding Gata4, Mef2c orTbx5 tagged with green fluorescent protein (GFP), or GFP alone were prepared from relevant plasmids by the Baylor College of Medicine Gene Vector Core, as previously described^4, 5, 10^. Plasmids for human ETV2 and reverse tetracycline-controlled transactivator (rtTA) were gifts from Dr. Rinpei Morita, Department of Microbiology and Immunology, Keio University, Tokyo, Japan. An adenovirus vector expressing VEGF (AdVEGF-All6A^+^) based on an Ad5 serotype backbone with deletions in the E1 and E3 regions and containing an artificial splice sequence cassette was prepared by the Belfer Gene Therapy Core Facility at Weill Cornell Medical College, New York, NY.

### Cell reprogramming

To assess cardio-differentiation efficiency, endothelial cells and cardiac fibroblasts cultured in EGM-2 medium supplemented with 10 ng/ml VEGF and bFGF were treated for 14 days with lentivirus encoding Gata4, Mef2c and Tbx5 (GMT) or GFP alone at a multiplicity of infection (MOI) of 20. To induce ETV2 expression, rat cardiac fibroblasts were treated with lentivirus encoding ETV2 and a second lentivirus encoding rtTA (MOI of 20 each) in EGM-2 medium supplemented with SingleQuots (cc-4176, Lonza). ETV2 was overexpressed in ETV2/rtTA-treated cells by doxycycline addition (100 ng/ml) into the cell culture media for a period of 10 days for rat cardiac fibroblasts, but for a period of only 3 days for human cardiac fibroblasts because of the rapid proliferation of these cells. As a control, naïve cells received only doxycycline. Three days after doxycycline removal, cells were treated with lentivirus encoding GMT (20 MOI) and maintained for 14 days in iCM medium (DMEM with 10% FBS and 20% M199).

For cell contractility studies, neonatal rat cardiomyocytes isolated from 0 to 3 days old neonatal rat pups were cultured DMEM and M199 in a 4:1 ratio and supplement with 10% horse serum, 5% fetal bovine serum, as previously described^15^. Adult rat cardiac fibroblasts treated with GFP-labeled reprogramming factors were harvested and re-plated onto cultures of neonatal rat cardiomyocytes at a ratio of 1:10 in DMEM/M199/10% FBS medium^12^.

### Fluorescence-activated cell sorting (FACS) analysis

For FACS analysis, cells were washed, trypsinized and fixed with fixation buffer (BD Biosciences, San Jose, CA) as previously described^5, 10^. Fixed cells were permeabilized with Perm/Wash buffer (BD Biosciences, San Jose, CA). For cTnT expression analysis, cells were incubated with primary cardiac troponin T (cTnT) antibody, (ab8295, 1:400) and secondary Alexa Fluor 647 (ab150107, 1:2000). For CD31 expression analysis, conjugated BV786 CD31 antibody (BD 744382, 1:100) was used. For CDH1 and CDH2 analysis, CDH1(ab76055,1:100) and CDH2 (ab18203, 1:100), secondary antibodies Alexa Fluor 405(ab175658, 1:2000) and Alexa Fluor 647 (ab150079, 1:2000) were used respectively. For human cells, primary (ab8295, 1:400) and secondary Alexa Fluor 647 (ab150107, 1:2000) antibodies were used for cTnT expression analysis. All analyses were performed by a LSR Fortessa cell sorter (BD Biosciences, Franklin Lakes, NJ) with FlowJo software (FlowJo, LLC, Ashland, Ore).

### qRT-PCR analysis

For qPCR studies, total RNA was extracted using TRIzol (Invitrogen) according to the vendor’s protocol. RNAs were then retro-transcribed to cDNA using iScript Supermix (Bio-Rad). qPCR was performed SYBR Green PCR Master Mix (Thermo Fisher Scientific) on a ViiA 7 Real-Time PCR System (Thermo Fisher Scientific). Results were normalized by comparative CT method with glyceraldehyde 3-phosphate dehydrogenase (GAPDH). All primer sequences are listed in Table 1.

**Table 1:** Primer sequences used for quantitative reverse transcriptase polymerase chain reaction.

Gene (rat)	Forward	Reverse
**cTnT**	AGGCTCACTTCGAGAACAGG	ATTGCGAATACGCTGCTGT
**Actc1**	GATTATTGCTCCCCCTGAGCG	GTGTAAGGTAGCCGCCTCAGAA
**Pln**	GTGACGATCACAGAAGCCAAGG	TGACAGCAGGCAGCCAAACG
**Gja1**	GAACAGTCTGCCTTTCGCTG	AAGGACCCAGAAGCGCACGT
**CD31**	CTCAGTCGGCTGACAAGATG	AGGCTTGCATAGAGCAGCAT
**CXCL12**	CCCTGCCGATTCTTTGAG	GCTTTTCAGCCTTGCAACA
**ESM1**	GGGGAAACCTGCTACCGTA	CTCCTTGCAATCCATCCCGAAC
**Gata4**	CCGGGCTGTCATCTCACTAT	GAGAGCTTCAGAGCCGACAG
**Mef2c**	GACAAGTACAGGAAAATTAACGAAGA	TGGGAGGTGGAACAGCAC
**Tbx5**	GCACAGAAATGATCATCACCAA	GGCCAGTCACCTTCACTTTG
**Twist**	AGCTACGCCTTCTCCGTCT	TCCTTCTCTGGAAACAATGACA
**Zeb1**	GCCAACAGACCAGACAGTGTT	CGCATTCGTCATCTTTTACG
**CDH1**	GGCTTGGATTTTGAGGCCAAGC	GCGATCTCCAGACCCACACC
**CDH2**	GGAAGCTGGCATCTATGAAG	CTCCATTGGAGTCACATTGGC
**GAPDH**	GGCACAGTCAAGGCTGAGAATG	ATGGTGGTGAAGACGCCAGTA
ETV2*	AGGGAACAAGCTGGCAGGGCTTGAA	TCCAGCATGTCTCTGCTGTCGCTGT

*Human.

### Immunofluorescence analysis

Immunofluorescence studies were performed after 4% paraformaldehyde fixation and permeabilization of cells with 0.5% Triton-X solution. Cells were then blocked with 10% goat serum and incubated with primary antibodies against cTnT (1:300 dilution; Thermo Fisher Scientific), α-actinin (1:400 dilution; Sigma-Aldrich, St. Louis, MO) and CD31 (1:400 dilution, Abcam ab64543). Goat anti-mouse Alexa 568 was used as the secondary antibody (1:1000 dilution; Thermo Fisher Scientific). Images were captured at the Core Fluorescence microscope and analyzed using ImageJ. To quantify marker-positive cells, the number of marker- positive cells and total cells quantified marked by DAPI (4’, 6-diamidino-2-phenylindole) staining were counted in three random images (10 × magnification) selected by an investigator blinded to treatment group.

### Measurements of contractility and calcium transient

Cell contractility (cell shortening) and calcium transients were measured in co-culture studies at room temperature. To perform these studies, cells were placed in a Plexiglas chamber which was positioned on the stage of an inverted epifluorescence microscope (Nikon Diaphot 200), and perfused with 1.8 mmol/L Ca^2+^‐Tyrode's solution containing (in mmol/L): NaCl 140, KCl 5.4, MgCl_2_ 1, CaCl_2_ 1.8, HEPES 5, and glucose 10, pH 7.4. Field-stimulation was provided by a Grass S5 stimulator using platinum electrodes placed alongside a cell culture bath containing 1.8 mM Ca^2+^, with bipolar pulses delivered at voltages 50% above myocyte stimulation thresholds. Contractions of iCMs from random fields were videotaped and digitized on a computer. For Ca^2+^ signal measurements, cells were loaded with 2 μmol/L of Fura‐2/AM (Life Technologies) and alternately excited at 340 and 380 nm at 0.5 Hz by use of a Delta Scan dual‐beam spectrophoto-fluorometer (Photon Technology International, Edison, NJ). Ca^2+^ transients were expressed as the 340/380‐nm ratios of the resulting 510‐nm emissions. Data were analyzed using Felix software (Photon Technology International)^10, 15^.

### Statistical analysis

Statistical analysis was performed using SAS version 9.2 (SAS Institute Inc, Cary, NC). Unpaired Student’s t-test or ANOVA was used for data analysis. Data are presented as the mean ± SEM, unless otherwise indicated. If ANOVA was significant for more than 2-group comparison, Bonferroni correction for ANOVA was followed for each pair comparison.

## Results

### Endothelial cells are more efficiently reprogrammed into cardiomyocyte-like cells than are cardiac fibroblasts

**Figure 1:**
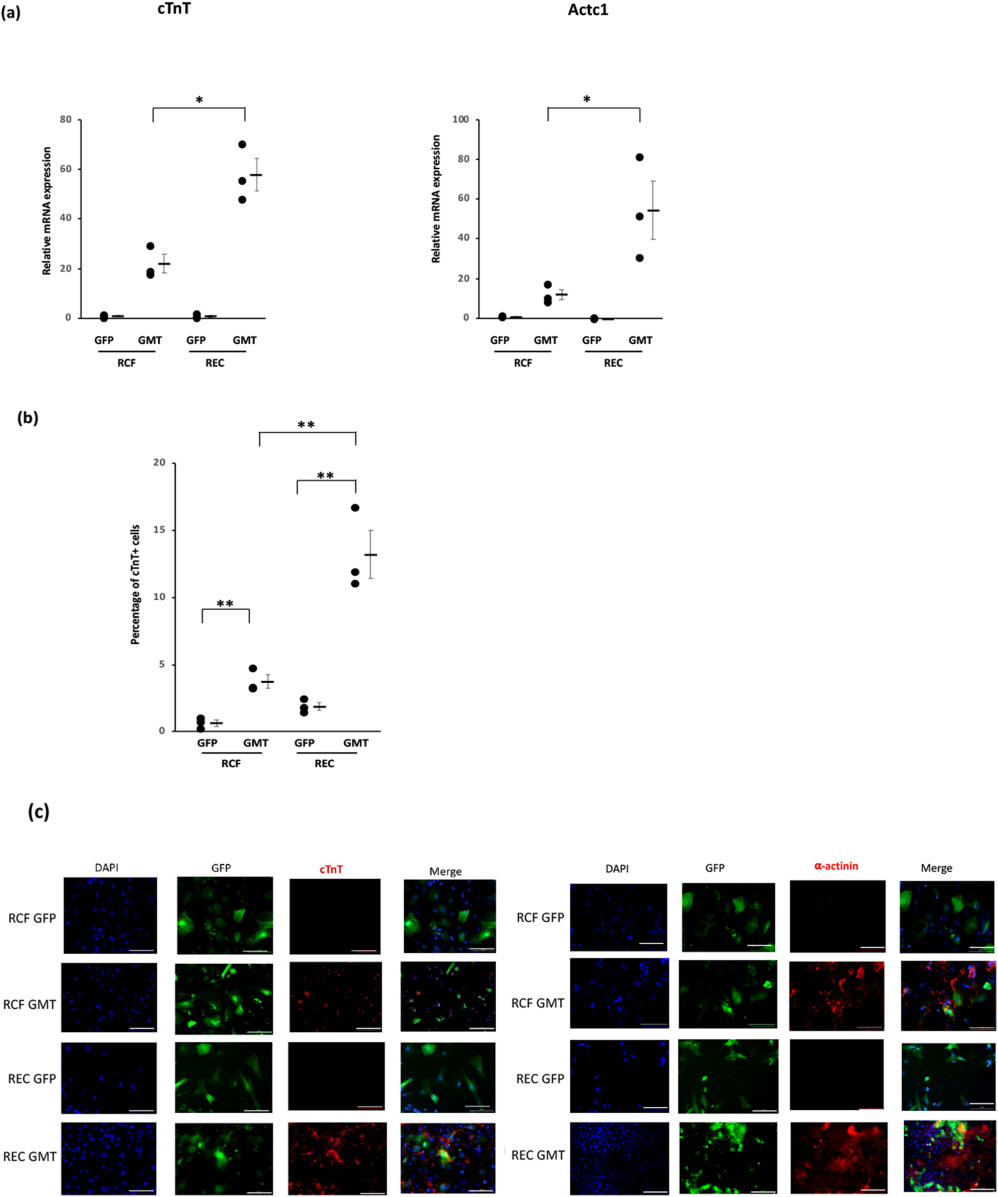
Endothelial cells are more efficiently reprogrammed into cardiomyocyte-like cells than are cardiac fibroblasts. Rat cardiac fibroblasts (RCF) and rat cardiac microvascular endothelial cells (REC) were treated with lentivirus encoding GFP or GMT (20 MOI) for 14 days (n = 3). (**a**) qPCR analysis demonstrating that cTnT and Actc1 expression was significantly increased in GMT-treated endothelial cells compared to GMT-treated cardiac fibroblasts (*p < 0.05). (**b**) Quantification of FACS data demonstrating an increased percentage of cTnT^+^ cells in GMT treated endothelial cells compared to GMT treated cardiac fibroblasts (**p < 0.01) (**c**) Immunostaining demonstrating the increased prevalence of cTnT^+^ and α-actinin^+^ cells in GMT-treated endothelial cells compared to GMT-treated cardiac fibroblasts. Scale bar: 100 μm.

qPCR analysis performed 14 days after rat cardiac fibroblasts and cardiac microvascular endothelial cells were infected with lentivirus encoding GMT demonstrated increased expression of the cardiomyocyte marker genes cTnT and Actc1 in GMT-treated endothelial cells vs GMT-treated cardiac fibroblasts (p < 0.05; Fig. 1a). FACS analysis likewise demonstrated that 13% ± 2% of endothelial cells treated with GMT expressed cTnT compared to 4% ± 0.5% of GMT-treated cardiac fibroblasts (p < 0.01; Fig. 1b). Immunofluorescence studies correspondingly demonstrated a greater proportion of cTnT and α-actinin positive cells in GMT-treated endothelial cells vs fibroblasts (Fig. 1c).

### ETV2 induces expression of endothelial cell markers in cardiac fibroblasts

**Figure 2:**
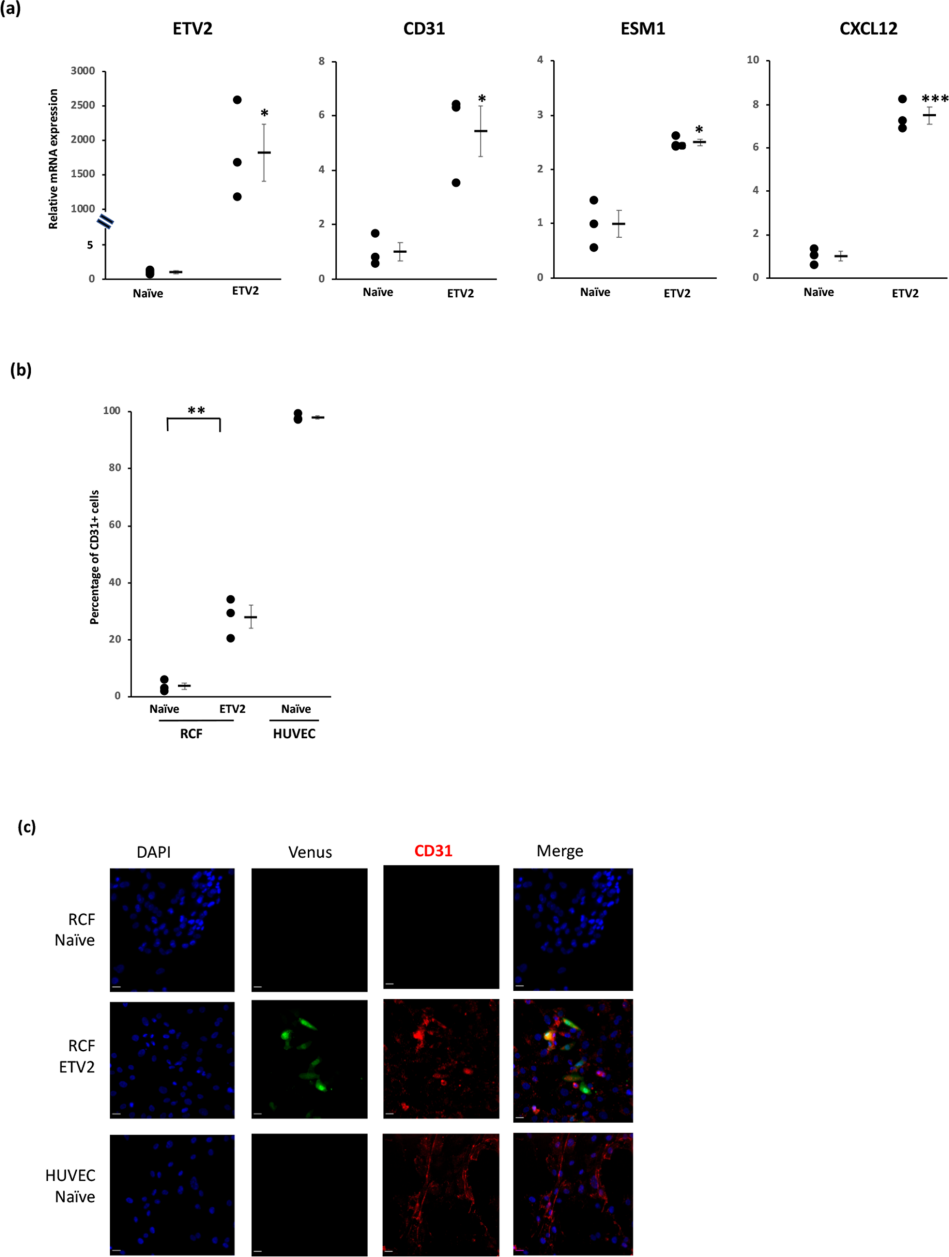
ETV2 induces expression of endothelial cell markers in cardiac fibroblasts. Rat cardiac fibroblasts were treated with doxycycline with or without administration of lentivirus encoding ETV2 and rtTA (n = 3). (**a**) qPCR analysis demonstrating upregulated expression of endothelial cell markers in ETV2-treated compared to naïve fibroblasts (*p < 0.05; ***p < 0.01). (**b**) Quantification of FACS data demonstrating an increased percentage of cells expressing endothelial cell marker CD31 in ETV2-treated compared to naïve fibroblasts (**p < 0.01). (**c**) Immunostaining demonstrating the presence of CD31^+^ cells in ETV2-treated compared to the absence of CD31^+^ expression by naïve fibroblasts. Scale bar: 200 μm.

qPCR analysis of rat cardiac fibroblasts induced to overexpress ETV2 demonstrated significantly increased expression of the endothelial cell markers CD31 (5.4-fold, p < 0.05), endothelial specific molecule-1 (2.5-fold, p < 0.05) and angiogenesis marker, C–X–C motif chemokine ligand 12 (7.5-fold, p < 0.001) compared to fibroblasts without induced ETV2 overexpression (Fig. 2a). FACS likewise demonstrated increased expression of the endothelial cell marker CD31 in ETV2-induced compared to non-induced fibroblasts (28% ± 4% vs 4% ± 2%, p < 0.01; Fig. 2b), as confirmed by immunofluorescence studies (Fig. 2c).

### ETV2 induction prior to GMT treatment enhances the reprogramming of rat cardiac fibroblasts into cardiomyocyte-like cells

**Figure 3:**
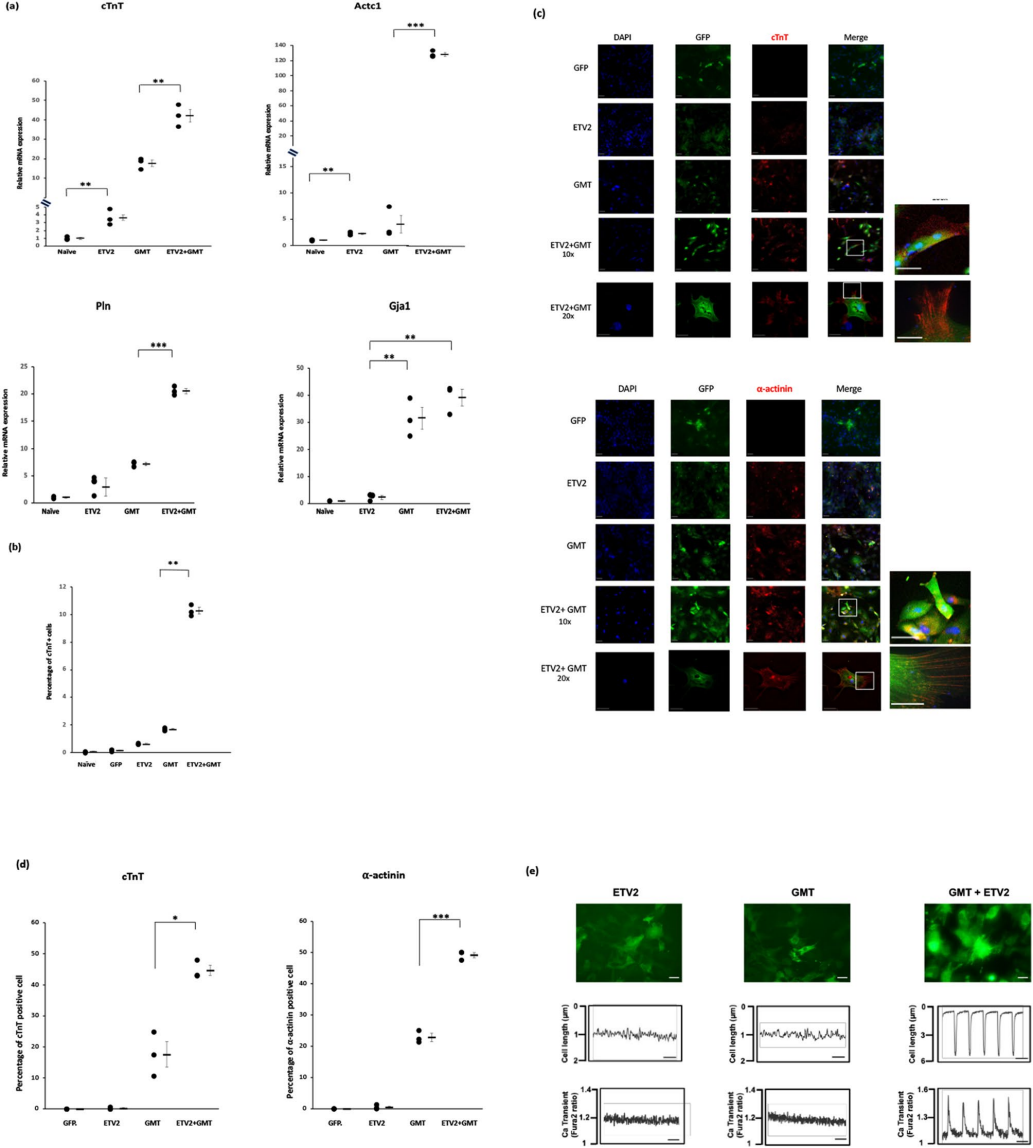
ETV2 induction prior to GMT treatment enhances the reprogramming of rat cardiac fibroblasts into cardiomyocyte-like cells. Rat cardiac fibroblasts were treated with doxycycline with or without lentivirus encoding ETV2 and rtTA. Three days after doxycycline withdrawal, cells were treated for 14 days with lentivirus encoding GFP or GMT (n = 3). (**a**) qPCR analysis demonstrating increased expression of the cardiomyocyte markers cTnT (**p < 0.01) as well as Actc1 and Pln (***p < 0.001) in ETV2 + GMT treated cardiac fibroblasts compared to cells treated with GMT alone. (**b**) Quantification of FACS data demonstrating an increased percentage of cTnT^+^ cells after ETV2 + GMT treatment vs treatment with GMT alone (**p < 0.01). (**c**) Immunostaining demonstrating the increased prevalence of cTnT and α-actinin in cells treated with ETV2 + GMT compared to cells treated with GMT alone. Sarcomeric filament structure was clearly identified in GMT + ETV2 groups but not in cells treated by GMT alone. High magnification views shown on the right side (top: × 40, bottom: × 100). Scale bar: 100 μm. (**d**) Quantification of immunostaining demonstrating increased number of cTnT-positive (*p < 0.05) as well as α-actinin-positive (***p < 0.001) cells in ETV2 + GMT treated cardiac fibroblasts compared to cells treated with GMT alone. (**e**) Cell contractility assessments of ETV2 (left), GMT (middle) and ETV2 + GMT (right) treated cells, as described in “Methods”. Two weeks after treatment with GFP-labeled reprogramming vectors, rat cardiac fibroblasts were transferred into co-culture with (untreated) neonatal rat cardiomyocytes (negative for GFP). Upper panel demonstrating GFP expression (green) of treated cells after 6 weeks in co-culture. Scale bar 100 μM. Lower panel demonstrating representative peaks from GFP-positive cells reflecting contraction (top row) and Ca^2+^ transients (bottom row) in cells treated with GMT + ETV2. Scale bar 0.5 Sec.

Rat cardiac fibroblasts overexpressing ETV2 demonstrated greater expression by qPCR of the cardiomyocyte markers cTnT and Actc1 compared to naive cells (cTnT, 3.7-fold, p < 0.01, Actc1, 2.3-fold, p < 0.01) (Fig. 3a). Rat cardiac fibroblasts treated with GMT after ETV2 induction (“ETV2 + GMT”) demonstrated significantly greater expression by qPCR of cTnT (2.4-fold increase, p < 0.01), Actc1 (32-fold increase, p < 0.001) and phospholamban (2.9-fold increase, p < 0 0.001) compared with fibroblasts treated with GMT alone (Fig. 3a). GMT administration led to equivalently increased expression of the cardiomyocyte marker Gja1 expression regardless of ETV2 expression (Fig. 3a).

FACS analysis likewise demonstrated that significantly more ETV2 + GMT treated rat cardiac fibroblasts expressed cTnT compared to cells treated by GMT or ETV2 alone (10% ± 0.2% vs 2% ± 0.1% and 0.6% ± 0.03%, respectively; p < 0.01; Fig. 3b). Immunocytochemistry confirmed the increased prevalence of cTnT and α-actinin positive cells following fibroblast treatment with ETV2 + GMT versus GMT treatment alone (Fig. 3c).

Quantification of cells positive for cTnT and α-actinin markers utilizing immunofluorescence-stained images showed that 45% ± 2% of the cells were cTnT positive in ETV2 + GMT treated cells compared to 18% ± 4% of the cells in GMT alone treated cells (p < 0.05) and 49% ± 1% of the cells were α-actinin positive in ETV2 + GMT treated cells compared to 23% ± 1% of the cells in GMT alone treated cells (p < 0.001) (Fig. 3d). Moreover, fibroblasts treated with ETV2 + GMT showed myosin filament structure, suggesting the generation of more mature iCMs by ETV2 pre-treatment of cells vs GMT treatment alone (Fig. 3c).

Although rat cardiac fibroblasts treated with GMT + ETV2 were not observed to contract independently after up to 8 weeks in culture, ∼ 3% of rat cardiac fibroblasts treated with GMT + ETV2 demonstrated contractility synchronous with surrounding cardiomyocytes after 6 weeks of co-culture with neonatal cardiomyocytes, as verified by their GFP expression (Fig. 3e; Supplemental video S1–S3). In comparison, cells treated with GMT or ETV2 alone failed to contract in co-culture experiments (Fig. 3e; Supplemental videos S1–S3). Cells treated with GMT + ETV2 also demonstrated calcium transients upon electrical stimulation that was synchronous with their contractile function, whereas calcium transients were not observed after stimulation of cells in other treatment groups (Fig. 3e).

### ETV2 induction prior to GMT treatment enhances the reprogramming of human cardiac fibroblasts into cardiomyocyte-like cells

**Figure 4:**
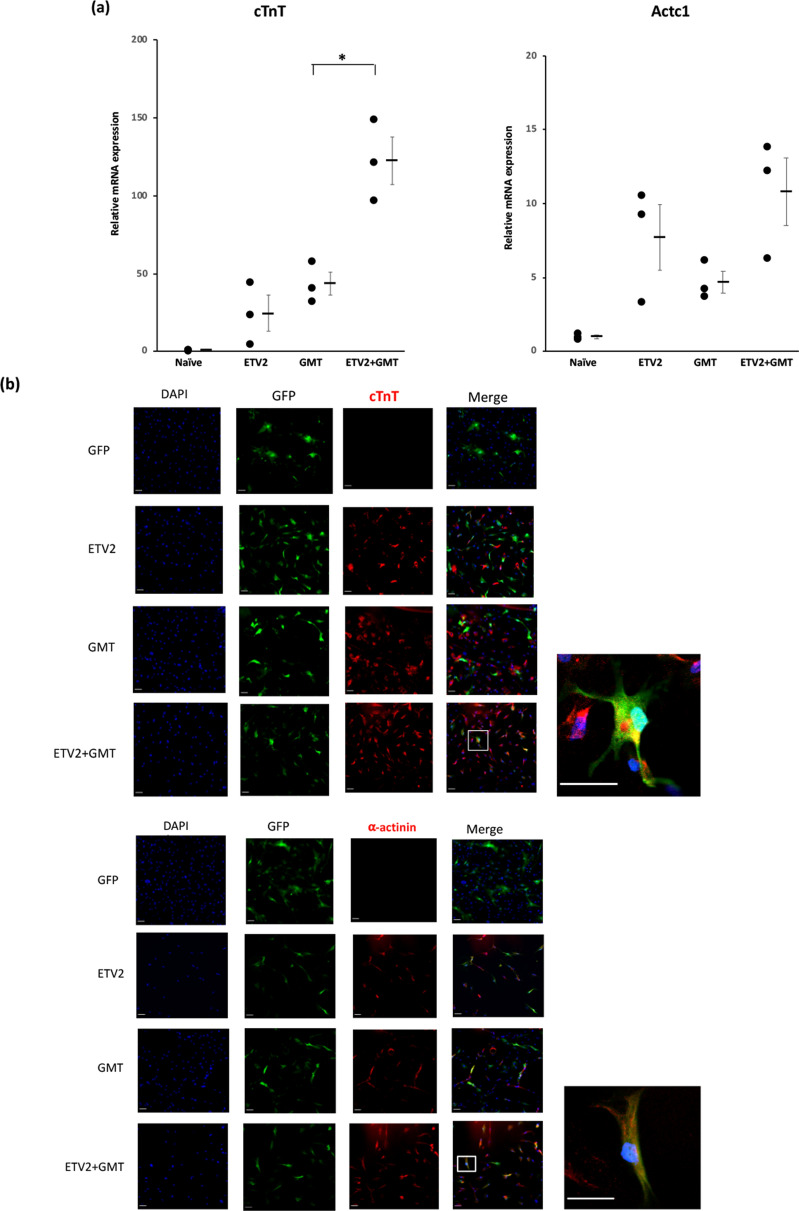
ETV2 induction prior to GMT treatment enhances the reprogramming of human cardiac fibroblasts into cardiomyocyte-like cells. Human cardiac fibroblasts were treated with doxycycline with or without administration of lentivirus encoding ETV2 and rtTA. Three days after doxycycline withdrawal, cells were treated for 14 days with lentivirus encoding GFP with or without GMT (n = 3). (**a**) qPCR analysis demonstrating increased expression of the cardiomyocyte markers cTnT (*p < 0.05) in ETV2 + GMT treated cardiac fibroblasts compared to cells treated with GMT alone. (**b**) Immunostaining demonstrating the increased prevalence of cTnT and α-actinin in cells treated with ETV2 + GMT compared to cells treated with GMT alone. High magnification views (× 100) are shown on the far right. Scale bar: 100 μm.

Human cardiac fibroblasts also demonstrated increased cardio-differentiation efficiency after ETV2 induction compared to cells treated with GMT alone. Specifically, qPCR analysis demonstrated that human cardiac fibroblasts treated with ETV2 + GMT expressed significantly higher cTnT compared to cells treated by GMT alone (2.8-fold increase, p < 0.05; Fig. 4a). Immunocytochemistry likewise demonstrated the increased prevalence of cTnT and α-actinin expression in human cardiac fibroblasts following treatment with ETV2 + GMT versus cells treated with GMT alone (Fig. 4b).

### ETV2 treatment induces cell plasticity and cardiogenic marker expression in cardiac fibroblasts

**Figure 5:**
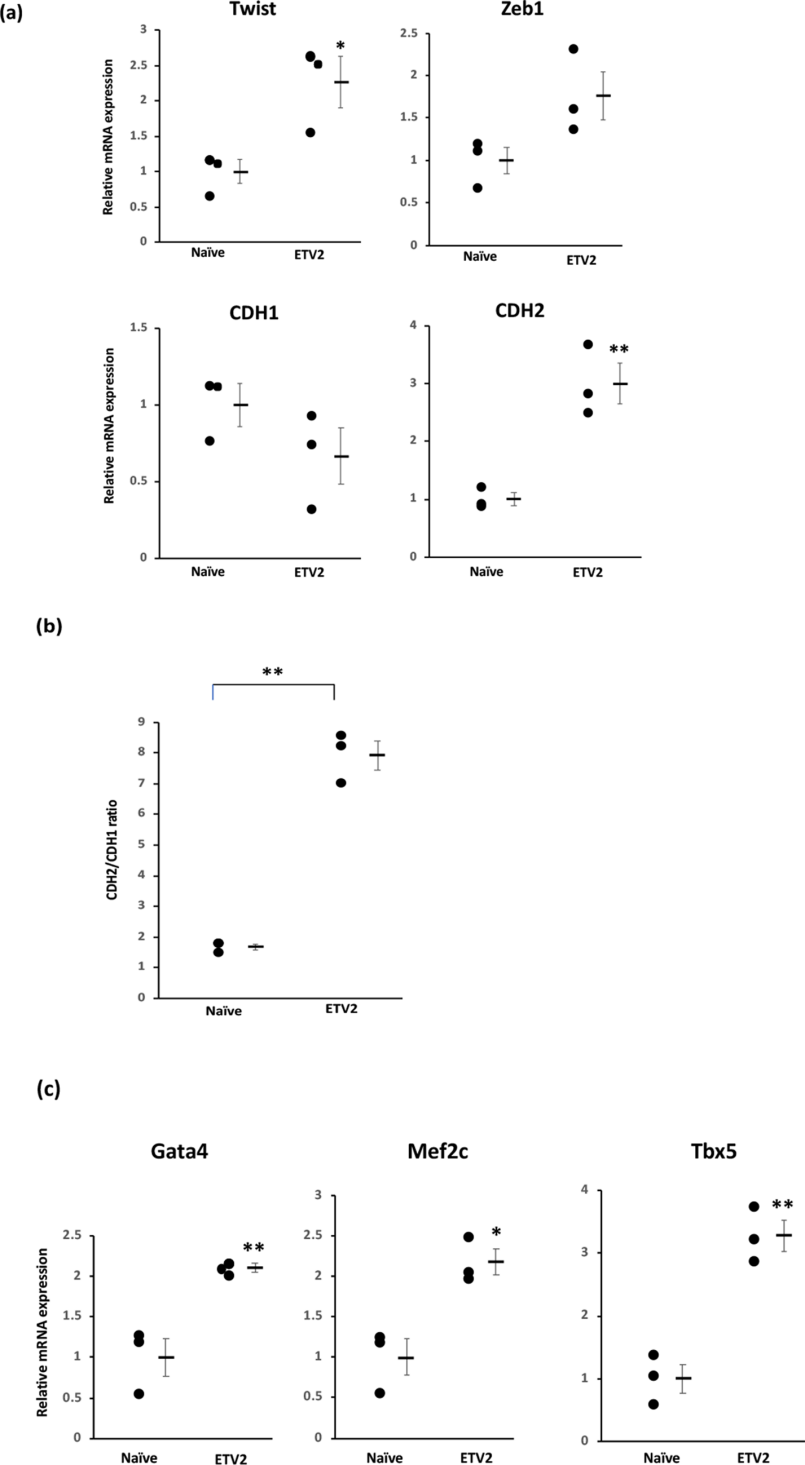
ETV2 treatment induces cell plasticity and cardiogenic marker expression in cardiac fibroblasts. Rat cardiac fibroblasts were treated with doxycycline with or without administration of lentivirus encoding ETV2 and rtTA (n = 3). (**a**) qPCR demonstrating upregulated expression of the endothelial-mesenchymal transition (EndMT) markers Twist (*p < 0.05) and CDH2 (**p < 0.01) in ETV2-treated compared to naïve fibroblasts. (**b**) Quantification of FACS data demonstrating an increased CDH2/ CDH1 ratio reflecting EndMT in cells overexpressing ETV2 (**p < 0.01). (**c**) qPCR demonstrating upregulated expression of cardiogenic markers Gata4, Mef2c and Tbx5 in ETV2-overexpressing compared to naïve fibroblasts (* p < 0.05; ** p < 0.01).

As a potential explanation for the enhanced cardio-differentiation potency of ETV2 + GMT treatment compared to cell treatment with GMT alone, we observed the increased expression of (pro-plasticity) EndMT pathway markers such as Twist (p < 0.05), Zeb1 (p < 0.08) and CDH2 (p < 0.01) in ETV2 overexpressing vs naïve rat cardiac fibroblasts, (Fig. 5a). FACS analysis likewise demonstrated increased CDH2^+^/CDH1^+^ ratio (indicator of EndMT) compared to naive fibroblasts (7.9 ± 0.5 vs 1.7 ± 0.1, p < 0.01; Fig. 5b). We also observed the upregulation of the cardiogenic genes Gata4 (p < 0.01), Mef2c (p < 0.05), and Tbx5 (p < 0.01) in rat cardiac fibroblasts overexpressing ETV2 compared to naïve cells (Fig. 5c).

## Discussion

Efforts to induce the direct transdifferentiation of fully differentiated adult cells have been spurred by the initial discovery by Yamanaka over a decade ago that adult somatic cells could be de-differentiated into induced pluripotent stem (iPS) cells and these could be re-differentiation into a wide variety of cell types^24^. Interestingly, the vast majority of these efforts have used mesenchymal cells, and fibroblasts in particular, as their starting cell target^25^. As the vista for such efforts has advanced to potential human applications, the relatively poor transdifferentiation efficiency of human cells have become increasingly problematic challenges to the advancement of this tissue regeneration strategy^26–29^.

The resistance of mature cells to reprogramming is believed to arise from greater epigenetic controls over (reprogramming) gene activation in higher versus lower order species^10, 26–31^. Work by our group and others suggest that “pro-plasticity” counter-strategies that could make target cells more susceptible to reprogramming may represent a useful approach to overcoming this hindrance, as opposed to the commonly used strategy of adding a greater number of factors to reprogramming cocktails^6–11, 15, 26–30^. In this paper, we specifically demonstrate that the pro-plasticity characteristics of the naturally occurring EndMT pathway can be leveraged to enhance cardiac fibroblast reprogramming into an induced cardiomyocyte phentotype, as evidenced by the enhanced susceptibility of endothelial vs fibroblast to cardio-differentiation. We demonstrate that preconditioning cardiac fibroblasts with ETV2 prior to GMT increased cardiomyocyte marker gene expression compared to cells treated with ETV2 or GMT alone. Importantly, about 3% of ETV2 + GMT treated cells demonstrated contractility when co-cultured with neonatal cardiomyocytes, reflecting a level of functional cardiodifferentiation.

The proposition of our EndMT pro-plasticity strategy is potentially hindered by the prevalence of fibroblasts, rather than endothelial cells, as the primary constituent of myocardial scar tissue, and as such have been the default target of post-infarct myocardial regeneration strategies. Our demonstration that the endothelial cell differentiation factor ETV2 can be used to transition fibroblasts into a “trans-endothelial cell” state addresses this challenge, successfully rendering a new target for standard cardiodifferentiation cocktails such as GMT^20–23^. Interestingly, our observation of cardiomyocyte marker expression in ETV2-treated fibroblasts even without GMT treatment suggests the potency of the EndMT pathway in driving cardio-differentiation. The teleological basis for the predilection of these cells towards such cardio-differentiation may be related to the primacy of cardiac tissue as the substrate of the first organ formation during embryologic development^32, 33^.

We used transient (Dox-inducible) ETV2 overexpression strategy in order to minimize the persistence of endothelial cell differentiation influences that could have acted in opposition to our cardiodifferentiation treatments. While we did not assess constitutive ETV2 expression to test this hypothesis, prior evidence that sustained ETV2 expression prevents cardiomyocyte differentiation and leads to a sustained endothelial cell phenotype during embryologic development supports the premise that a “trans-endothelial” state induced by transient ETV2 overexpression represents a cell target preferable for cardio-differentiation^34^.

Interestingly, our current findings are potentially consistent with that of our prior investigations demonstrating that administration of angiogenic vascular endothelial growth factor (VEGF) to infarcted myocardium prior to GMT administration enhances the efficacy of GMT in improving myocardial function compared with use of GMT alone^4^. In these studies, we demonstrated that VEGF induces angiogenesis in the treated infarcted myocardial and presumed that VEGF thus acted to “pre-vascularize” the scar tissue and thereby support the survival of subsequently induced cardiomyocytes in the infarct milieu. In recent in vitro studies, we have however now demonstrated that like ETV2, VEGF pre-treatment increases the cTnT expression of GMT-treated fibroblasts (Supplemental Fig. 1). These findings discount our “pre-vascularization” model as the only potentially cause of the enhanced reprogramming effect of VEGF. Given its established human clinical safety record^35, 36^, and evidence that it may also induce endothelial differentiation^37^, VEGF may thus represent an important potential mediator of our “trans-endothelial” strategy in the clinical setting.

In the context of our prior suppositions regarding enhancement of the effects of cardiac reprogramming by VEGF in vivo, it is interesting that Lee et al. have also noted that administration of ETV2 at the time of coronary ligation improves post-MI cardiac function, although they attributed their observation to ETV2-mediated endothelial cell proliferation and angiogenesis rather than to cardiac fibroblast—endothelial cell transdifferentiation^38^. In contrast to this observation, which may have salvaged ischemic border zones from progressing to infarction, we did not observe an improved post-infarct cardiac function with VEGF treatment 3 weeks after coronary ligation, likely because of the lack of viable cardiomyocytes in infarcted ventricles^4, 39, 40^. These studies both thus lend support to the premise of adding angiogenic/trans-endothelial to cardiodifferentiation strategies to salvage infarcted myocardium.

Our proposed trans-endothelial strategy poses the theoretical risk of excessive endothelial cell generation and vasculature deformation or even hemangioma formation, as previously shown with prolonged administration of angiogenic mediators^41^. This potential risk should be addressed by our proposed transient ETV2 expression strategy, which could be provided by regulatable transgenes or limited expression vectors. Assuming comparable effects of VEGF and ETV2, the absence of such effects induced by VEGF in animal and human studies likewise speaks against the likelihood of this concern^42, 43^. The theoretical risk of ETV2 inducing dystopic influences on the vasculature could also be overcome by the incorporation of fibroblast specific promoters in ETV2 vectors.

Taken together, this study demonstrated that endothelial cells and cardiac fibroblasts transitioned into an endothelial cell “trans” state can be transdifferentiated into iCM cells with higher efficiency than are fibroblasts not exposed to such interventions. This alternative to a traditional fibroblast-directed strategy may represent an important new approach to cardiac cell reprogramming and post-infarct myocardial regeneration in clinical post-infarct therapies.

### Study limitations

We have assumed that the pro-plasticity propensities of ETV2-mediated reprogramming of fibroblasts relates to effects of endothelial cell transdifferentiation and/or EndMT pathway activation**.** Recent articles suggest however, that ETV2 may also induce global epigenetic changes that may represent an alternative or supplemental pro-plasticity pathway^44, 45^. We plan to perform additional analysis of global epigenetic changes such as RNAseq, ATACseq and ChIPseq to further investigate the implications of such mechanisms. In this context, it would be interesting if we are able to identify a specific subset of cardiac fibroblasts more susceptible to entering a “pro-plasticity” endothelial cell trans state. We are planning cell lineage tracing studies to further explore this avenue of investigation.

## Supplementary Information

Supplementary Legends.

Supplementary Figure 1.

Supplementary Video 1.

Supplementary Video 2.

Supplementary Video 3.

## Abbreviations

Actc1: Actin alpha cardiac muscle 1
cTnT: Cardiac troponin T
CXCL12: C-X-C motif chemokine ligand 12
ESM1: Endothelial specific molecule 1
ETV2: ETS variant transcription factor 2
FACS: Fluorescence-activated cell sorting
FBS: Fetal bovine serum
GFP: Green fluorescent protein
GJA1: Gap junction protein alpha1
GMT: Gata4, Mef2c and Tbx5
HUVEC: Human umbilical vein endothelial cell
iCM: Induced cardiomyocyte-like cell
Pln: Phospholamban
RCF: Rat cardiac fibroblast
REC: Rat cardiac microvascular endothelial cell
