# Supplementary material for: Fibroblast transition to an endothelial “trans” state improves cell reprogramming efficiency
Source: Sci Rep. 2021 Nov 19;11:22605. doi: 10.1038/s41598-021-02056-x (PMC8604927; doi:10.1038/s41598-021-02056-x)
Supplement: Supplementary file 1 — Supplementary Legends. [file 41598_2021_2056_MOESM1_ESM.docx]

**Transition to an Endothelial “Trans” State Improves Cell Reprogramming Efficiency**

Megumi Mathison, Deepthi Sanagasetti, Vivek P. Singh, Aarthi Pugazenthi, Jaya Pratap Pinnamaneni, Christopher T. Ryan, Jianchang Yang, Todd K. Rosengart

**Supplemental Figure Legend**

**Figure 1. VEGF pre-treatment enhances iCM reprogramming *in vitro*.** Rat cardiac fibroblasts were treated for 10 days with adenovirus encoding VEGF (MOI 20) or with lentivirus encoding ETV2 and rtTA together (MOI of 20 each) with doxycycline for 10 days (n=3). Three days after doxycycline removal, all cells were treated for 14 days with lentivirus encoding GMT. qPCR analysis demonstrating 2.0-fold increase in cTnT expression in VEGF+GMT treated cardiac fibroblasts and 2.2-fold increase in ETV2+GMT treated cardiac fibroblasts compared to the cells with GMT alone. *p < 0.05, **p < 0.01.

**Videos S1, S2, and S3. ETV2 + GMT treatment induces contractile iCMs.** Cell contractility was assessed between ETV2, GMT and ETV2+GMT treated cells. Two weeks after GMT treatment, these rat cardiac fibroblasts were co-cultured with (untreated) neonatal rat cardiomyocytes (negative for GFP).

**Video S1.** This video shows rat cardiac fibroblasts treated with an ETV2 vector did not demonstrate contraction.

**Video S2.** This video shows rat cardiac fibroblasts treated with a GMT vector did not demonstrate contractions.

**Video S3.** This video shows rat cardiac fibroblasts treated with ETV2 and GMT vectors contracted synchronously with surrounding rat cardiomyocytes.
